# Comparative evaluation of accuracy, precision, and radiation dose between mindways and low-dose iCare QCT for lumbar spine BMD using the European spine phantom

**DOI:** 10.3389/fmedt.2025.1575553

**Published:** 2025-05-12

**Authors:** Yujie Li, Tiantian Yin, Qiushi Yang, Heli Han, Zeguo Wang, Wanjiang Yu

**Affiliations:** ^1^ School of Medical Imaging, Shandong Second Medical University, Weifang, Shandong, China; ^2^ Department of Radiology, Qingdao Hospital, University of Health and Rehabilitation Sciences (Qingdao Municipal Hospital), Qingdao, Shandong, China

**Keywords:** bone mineral density, European spine phantom, quantitative CT, radiation dose, accuracy

## Abstract

**Background:**

Quantitative computed tomography (QCT) has received growing attention for its utility in bone mineral density (BMD) assessment and osteoporosis diagnosis.

**Objective:**

To assess the accuracy and precision of lumbar spine BMD measurements obtained using low-dose iCare QCT, based on the European Spine Phantom (ESP).

**Methods:**

Paired *t*-test was employed to compare BMD values measured under normal-dose and low-dose scan protocols using Mindways and iCare QCT systems. Accuracy was evaluated using relative measurement error (RME), and precision was assessed via relative standard deviation (RSD). Pearson correlation coefficients and Bland-Altman analysis were used to examine measurement correlation and agreement.

**Results:**

For Mindways QCT, RMEs of L1–L3 were 11.89%, 6.94%, and 6.72% under normal-dose, and 6.65%, 10.5%, and 6.31% under low-dose, respectively. For iCare QCT, RMEs were 1.21%, 4.28%, and 8.88% under normal-dose, and 2.14%, 4.96%, and 8.59% under low-dose, respectively. RSDs of L1–L3 for Mindways QCT were 5.16%, 2.85%, and 0.47% under normal-dose, and 9.08%, 4.69%, and 0.49% under low-dose, respectively. For iCare QCT, RSDs were 1.11%, 0.81%, and 0.45% under normal-dose, and 2.34%, 0.85%, and 0.33% under low-dose, respectively. The radiation dose in the low-dose protocol was significantly reduced compared with the normal-dose protocol.

**Conclusion:**

Low-dose iCare QCT exhibited high accuracy and precision in measuring lumbar spine BMD, achieving an approximately 85% reduction in radiation dose. These findings highlight its potential as a safer and reliable tool for clinical application.

## 1 Introduction

With the accelerated aging of the Chinese population, osteoporosis and osteoporotic fractures have become important public health issues (1, 2). Statistics have shown that the prevalence of vertebral fracture among people aged 40 and above in China is 10.5% and 9.7% for men and women, respectively (3). Bone mineral density (BMD) is an important indicator of bone quality and a reliable basis for screening and diagnosing osteoporosis and predicting osteoporotic fractures (4). The rapidly advancing quantitative computed tomography (QCT) technique has garnered more and more attention in the field of BMD measurement and osteoporosis diagnosis. Compared with dual x-ray absorptiometry (DXA), a key limitation of QCT is its relatively higher radiation dose. The iCare QCT bone densitometry system, a newly developed phantom-based QCT platform in China, has demonstrated preliminary advantages in affordability and measurement accuracy. Liu et al. (5) found that conventional normal-dose iCare QCT demonstrated a higher detection rate for osteoporosis compared to Mindways QCT; however, evidence regarding vertebral BMD measurements using low-dose iCare QCT remains limited.

Osteoporosis is a systemic skeletal disease characterized by low bone mass and microarchitectural deterioration, primarily affecting the vertebral bodies due to their high trabecular content (6, 7). Accurate and standardized measurement of BMD, especially in the spine, is essential for early diagnosis and treatment evaluation. However, in clinical and research settings, variability in imaging equipment, protocols, and analysis software can lead to inconsistencies in BMD assessment. To mitigate this, calibration phantoms are frequently employed to provide reference standards for quantitative imaging.

DXA remains the most commonly used method for BMD assessment due to its low cost and minimal radiation exposure; however, it provides only areal BMD and is limited by its inability to distinguish cortical from trabecular bone (5–7). In contrast, QCT allows for volumetric BMD measurements and separate evaluation of trabecular bone, which is more metabolically active and sensitive to early changes in bone mass (8–10). This makes QCT particularly valuable in detecting osteoporosis and monitoring treatment response. To reduce patient radiation exposure while maintaining diagnostic utility, the development of low-dose QCT protocols has become a key research focus (11, 12). However, dose reduction may compromise image quality and quantitative accuracy, making it essential to validate the performance of low-dose systems against established standards. Mindways QCT is a widely used and validated commercial system for spinal BMD assessment, and has served as a reference in both clinical and experimental studies (13). Comparative evaluations of different QCT platforms and scanning protocols are essential to ensure measurement consistency, particularly when implementing new systems or adjusting dose settings.

Although this study ultimately concentrated on the measurement accuracy and consistency of BMD values derived from a calibration phantom rather than human vertebral bodies, the implications of these findings are relevant for clinical vertebral BMD assessment. The European Spine Phantom (ESP) is a standardized calibration device designed to simulate human lumbar vertebrae in both geometry and BMD distribution (8, 9). It consists of three vertebral-shaped structures with known hydroxyapatite concentrations, enabling precise calibration of imaging systems (10, 11, 14). The ESP was selected for this study due to its widespread acceptance in clinical and research applications, as well as its ability to reduce inter-scanner and inter-site variability, making it an ideal benchmark for evaluating quantitative imaging performance.

## 2 Objective

This study aimed to compare the accuracy, precision, and repeatability of vertebral BMD measurements among low-dose iCare QCT, normal-dose iCare QCT, and Mindways QCT. The ESP was used to standardize measurements and assess whether radiation dose reductions with low-dose iCare QCT could be achieved without compromising diagnostic performance.

## 3 Materials and methods

### 3.1 Materials

According to the American College of Radiology guidelines for QCT, trabecular BMD values below 80 mg/cm^3^ are indicative of osteoporosis, values between 80 and 120 mg/cm^3^ represent osteopenia, and values above 120 mg/cm^3^ are considered normal bone density. ESP (serial number ESP-040; QRM GmbH, Moehrendorf, Germany) was used in this study. The phantom contained three different hydroxyapatite (HAP) inserts with BMDs of 50 mg/cm^3^ (0.506 g/cm^2^), 102 mg/cm^3^ (1.012 g/cm^2^), and 197 mg/cm^3^ (1.526 g/cm^2^), denoted as L1, L2, and L3, which represent osteoporosis, osteopenia, and normal bone mass, respectively.

### 3.2 Instrumentation and scanning method

**Figure 1:**
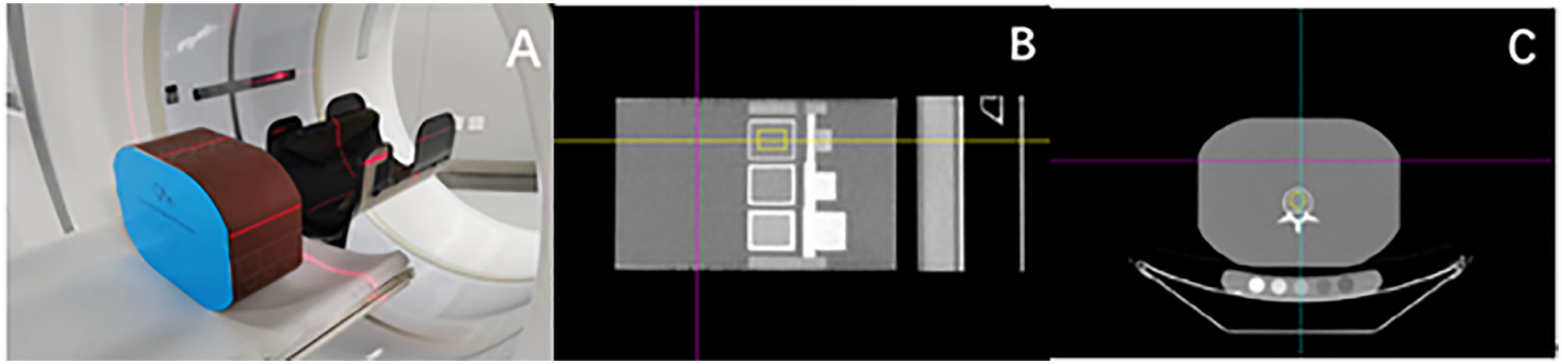
Quantitative CT measurement of BMD in ESP vertebrae. **(A)** is a schematic diagram for the scanning of ESP. **(B,C)** are schematic diagrams for the ESP vertebrae BMD measurement by iCare QCT post-processing software, with the yellow area being the ROI.

The ESP was scanned using a GE Revolution 256-row CT scanner (Revolution CT, GE Healthcare, Waukesha, WI, USA), with the ESP placed at the center of the scanning bed and a bed height of 172 cm, as shown in Figure 1A. The phantom was scanned using respective QCT protocols, and the scans were categorized into normal-dose and low-dose groups based on tube current. Conventional normal-dose QCT scan parameters: tube current: automatic milliampere-second technique (200–370 mA), tube voltage: 120 kV, detector width: 80 mm, pitch: 0.992:1, tube rotation speed: 0.8 s/r; the tube current in the low-dose group was 40 mA, and the rest of the parameters were the same. The thin-section (1.25 mm) images were reconstructed at the end of scanning and uploaded to the Mindways QCT workstation (Mindways Software Inc., Austin, TX, USA) and the iCare QCT workstation (iCare QCT, serial no. 1-01033; Hunan Junlang Technology Co., Ltd., Hunan, China). The volume CT dose index (CTDIvol) and dose length product (dose length product, DLP) produced by each scan of the CT scanner were recorded. The diameter of the ROI was set by the software algorithm based on vertebral body dimensions and phantom specifications, with an average diameter of approximately 10 mm. The ROIs were centered to avoid the cortical bone, vertebral edges, and the posterior venous plexus. No manual adjustments were made after automatic ROI generation, and all measurements were conducted through fully automated procedures to minimize operator-dependent variability. The images were post-processed using the built-in analysis modules of Mindways QCT and iCare QCT software, which automatically generated circular regions of interest (ROIs) at the central trabecular region of each vertebra (L1–L3). Both QCT measurement software programs were subjected to periodic calibration with the phantom to ensure the accuracy of the measurement results. Specifically, the Mindways QCT system was calibrated daily using the Mindways Model 3 CT calibration phantom (Mindways Software Inc.), containing reference rods with known hydroxyapatite concentrations for standardized BMD quantification. In contrast, the iCare QCT system underwent weekly calibration using its proprietary iCare QCT calibration phantom (Hunan Junlang Technology Co., Ltd.), which was designed with internal reference standards. The differing calibration intervals between the two systems were determined according to manufacturer guidelines. However, this inconsistency in calibration frequency could contribute to measurement variability. The scan was repeated 10 times for each group to assess precision and reduce variability, and the 10 repeated scans for each dose group were conducted consecutively during the same session to assess intra-observer repeatability under consistent conditions. All scans and post-processing procedures were performed by a single radiologist with over 7 years of experience in quantitative CT analysis to minimize inter-operator variability. For intra-observer repeatability assessment, all scans were performed and analyzed by a single radiologist with more than 7 years of experience in QCT imaging. To evaluate inter-observer repeatability, a second radiologist independently reprocessed the same set of images using identical software settings and ROI placement criteria. The BMD measurements from both observers were then compared statistically to assess consistency.

The automatic generation of ROIs in both QCT software platforms was based on proprietary algorithms that detect the central trabecular region of each vertebral body. These algorithms utilize a combination of vertebral geometry detection, Hounsfield unit (HU) thresholds, and phantom-specific positioning data to identify the mid-axial slice of each vertebra and delineate a circular ROI centered in the trabecular compartment, avoiding cortical bone and venous plexuses. The average ROI diameter was approximately 10 mm, although slight variations (±0.5 mm) could occur due to minor differences in vertebral shape or image contrast, especially under low-dose scan conditions. No manual intervention was performed to adjust the ROI location or size after initial detection. To assess the reliability of automated ROI placement, both intra- and inter-observer repeatability analyses were performed using repeated scans and reprocessing, as described below. Despite the fully automated nature of ROI detection, slight inter-software variability in ROI positioning could contribute to differences in BMD measurements, particularly under altered dose conditions or across software platforms.

### 3.3 Statistical analysis

Data were analyzed using SPSS 26.0 software (IBM Corp., Armonk, NY, USA). Quantitative data were tested for normality using the Shapiro–Wilk test and expressed as mean ± standard deviation (SD). Homogeneity of variances was assessed using Levene's test. For comparisons of measured BMD values across the four groups for each vertebra (normal-dose and low-dose scans using Mindways QCT and iCare QCT), one-way analysis of variance (ANOVA) was performed when both normality and homoscedasticity assumptions were met, followed by Tukey's *post hoc* test for pairwise comparisons. When these assumptions were violated, the Kruskal–Wallis test was applied instead, with Dunn's *post hoc* test and Bonferroni correction used for multiple comparisons.

Relative measurement error (RME) and relative standard deviation (RSD) were calculated to evaluate the accuracy and precision of measured BMD values. Comparisons of RME and RSD across groups were performed using one-way ANOVA or Kruskal–Wallis test, as appropriate based on data distribution and variance equality, followed by the corresponding *post hoc* analyses. Given the fixed structure and homogeneity of the phantom, 10 repeated measurements were regarded sufficient to evaluate repeatability. To further support reliability, 95% confidence intervals of Pearson correlation coefficients were reported. For iCare QCT scans, correlations between measured and true values, as well as between normal-dose and low-dose measurements, were assessed using Pearson correlation analysis. Agreement between normal-dose and low-dose measurements was further evaluated using Bland-Altman analysis. Statistical significance was set at *P* < 0.05.

The relative mean error (RME) was calculated as:

$$\mathrm{RME}\;(\text{\%})\;=\;[(\mu_{1}\;\text{-}\;\mu_{2})\;/\;\mu_{2}]\;\times 100,$$

where μ_1_ is the mean BMD value measured by the QCT system and μ_2_ is the known reference BMD value of the phantom insert. The relative standard deviation (RSD) was calculated as:

$$\mathrm{RSD}\;(\text{\%})=(\sigma /\mu_{1})\;\times 100,$$

where σ is the standard deviation of the repeated BMD measurements and μ_1_ is their mean.

## 4 Results

### 4.1 Differences in measured BMD values by the two types of QCT under different scan modes

**Figure 2:**
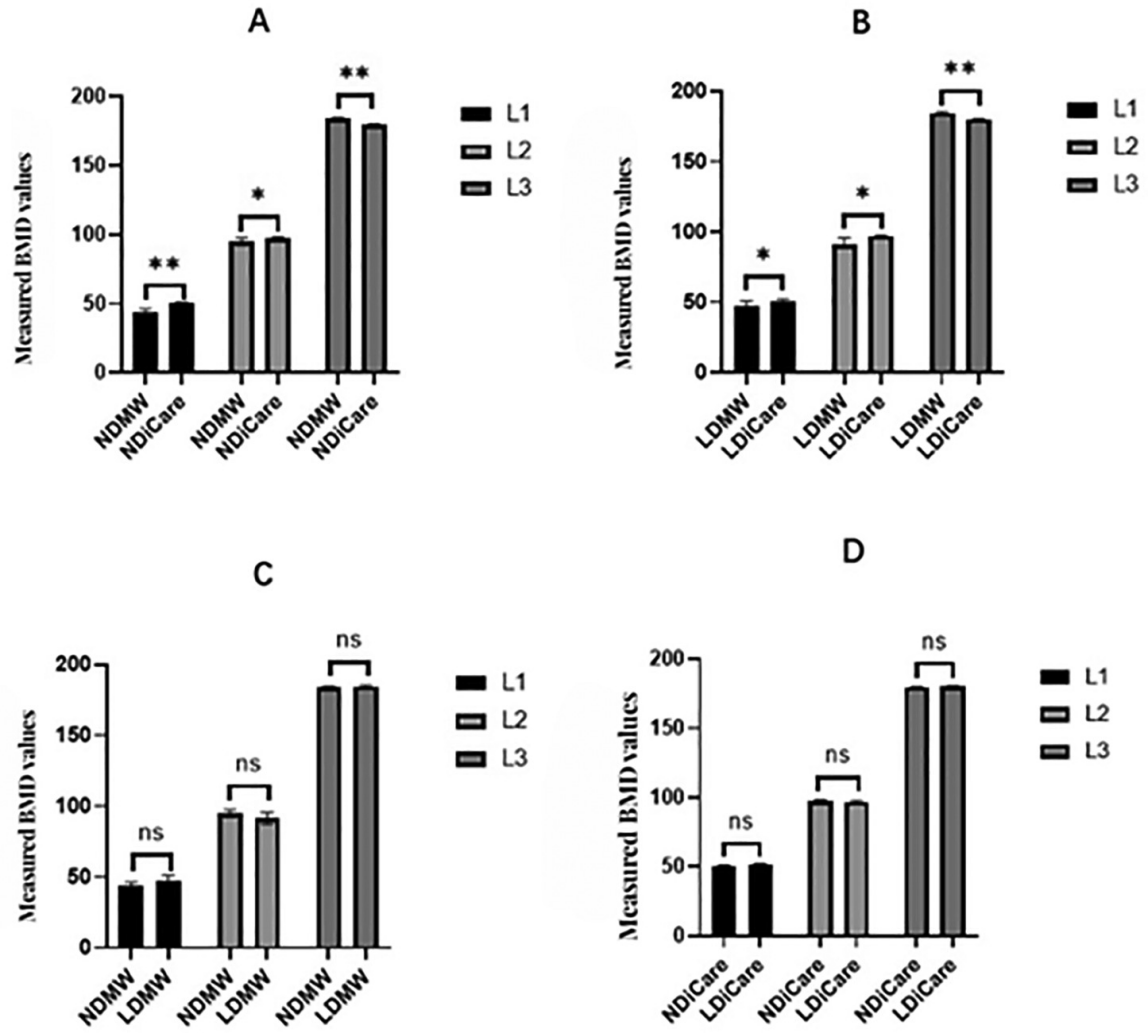
Comparison of measured BMD values (mg/cm^3^) for vertebral phantom inserts (L1–L3) across different scan modes and QCT software systems. **(A)** Comparison between normal-dose Mindways (ND/MW), normal-dose iCare (ND/iCare), low-dose Mindways (LD/MW), and low-dose iCare (LD/iCare). **(B)** Comparison between low-dose and normal-dose iCare. **(C)** Comparison between normal-dose and low-dose Mindways. **(D)** Comparison between normal-dose and low-dose iCare. Data are presented as mean ± SD. One-way ANOVA followed by Tukey's *post hoc* test was used when normality and homoscedasticity were confirmed; otherwise, the Kruskal–Wallis test followed by Dunn's *post hoc* test and Bonferroni correction was applied. *P < 0.05, **P < 0.01, ns, not significant. Sample size: *n* = 10 per group. NDMW, normal-dose Mindways QCT; LDMW, low-dose Mindways QCT; NDiCare, normal-dose iCare QCT; LDiCare, low-dose iCare QCT.

As presented in Figure 2A, significant differences were found in measured BMD values for all three vertebral inserts (L1–L3) between the normal-dose Mindways QCT and iCare QCT systems (*P* < 0.010), and between low-dose Mindways and iCare (*P* < 0.050), based on ANOVA with Tukey's *post hoc* test.

In contrast, no significant differences were found between normal-dose and low-dose scans within the same software platform for either Mindways (Figure 2C) or iCare (Figure 2D), as determined by the Kruskal–Wallis test (*P* > 0.050).

For the iCare QCT system, as shown in Figure 2B, measured values under normal-dose scanning were significantly higher than under low-dose scanning for L1 and L2 (*P* < 0.050 or *P* < 0.010), while differences for L3 were not statistically significant. These results suggest that BMD values measured by the two systems differ significantly under equivalent scanning conditions, while intra-system repeatability remains stable across dose levels.

### 4.2 RME (%) of measured BMD values by the two types of QCT under different scan modes

**Figure 3:**
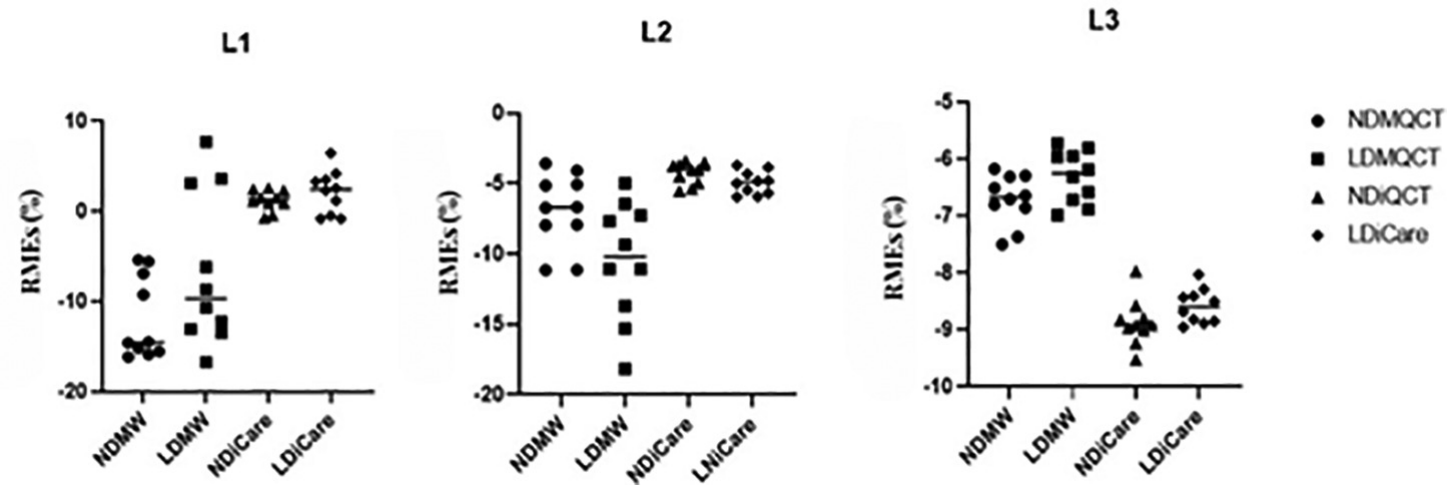
RMEs (%) of vertebrae BMD values measured by the two QCT software programs. NDMW, normal-dose Mindways QCT; LDMW, low-dose Mindways QCT; NDiCare, normal-dose iCare QCT; LDiCare, low-dose iCare QCT.

The RMEs of Mindways QCT measurements for L1–L3 were 11.89%, 6.94%, and 6.72%, respectively at normal-dose and 6.65%, 10.5%, and 6.31%, respectively at low-dose. The RMEs of iCare QCT measurements for L1–L3 were 1.21%, 4.28%, and 8.88%, respectively at normal-dose and 2.14%, 4.96%, and 8.59%, respectively at low-dose. The abovementioned results indicated that the RMEs of normal-dose and low-dose iCare QCT measurements for L1 and L2 were both smaller than the Mindways QCT measurements (Figure 3). Table 1 presents RME (%) by QCT system, scan mode, and vertebra.

**Table 1:** Relative measurement error (RME, %) by QCT system, scan mode, and vertebra.

Vertebra	Mindways (normal)	Mindways (low)	iCare (normal)	iCare (low)
L1	11.89%	6.65%	1.21%	2.14%
L2	6.94%	10.50%	4.28%	4.96%
L3	6.72%	6.31%	8.88%	8.59%

### 4.3 RSD (%) of measured BMD values by the two types of QCT under different scan modes

The RSDs of Mindways QCT measurements for L1–L3 were 5.16%, 2.85%, and 0.47%, respectively at normal-dose and 9.08%, 4.69%, and 0.49%, respectively at low-dose. The RSDs of iCare QCT measurements for L1–L3 were 1.11%, 0.81%, and 0.45%, respectively at normal-dose and 2.34%, 0.85%, and 0.33%, respectively at low-dose. The above results showed that the RSDs of iCare QCT measurements for L1–L3 at either normal-dose or low-dose were smaller than those of Mindways QCT; the RSDs of ESP vertebrae measurements by the two QCT software programs both decreased with increasing BMD. Although the number of repeated scans per group was limited to 10, the RSD values, particularly those from iCare QCT, were consistently low across all conditions (≤2.34%), indicating high repeatability of measurements. The low variability reflects the stable characteristics of the phantom and the consistency of the scanning protocol. Table 2 presents RSD (%) of BMD measurements.

**Table 2:** Relative standard deviation (RSD, %) of BMD measurements.

Vertebra	Mindways (normal)	Mindways (low)	iCare (normal)	iCare (low)
L1	5.16%	9.08%	1.11%	2.34%
L2	2.85%	4.69%	0.81%	0.85%
L3	0.47%	0.49%	0.45%	0.33%

BMD, bone mineral density.

### 4.4 Assessment of correlation and agreement between measurements by the two types of QCT under different scan modes

The BMD values measured by normal-dose and low-dose iCare QCT were highly correlated with the mean true BMD values (*r* = 0.99, 95% CI: 0.98–0.99, *P* < 0.001; *r* = 0.99, 95% CI: 0.98–0.99, *P* < 0.001) and the correlation coefficients between them showed no statistically significant difference (*P* = 0.202). Overall correlation analysis showed a high level of correlation between BMD values measured by normal-dose and low-dose iCare QCT (*r* = 0.99, 95% CI: 0.98–0.99, *P* < 0.0001). The Bland-Altman analysis showed that, for measured BMD values of L1–L3 by normal-dose and low-dose iCare QCT, the mean difference (d) was −0.11 mg/cm^3^, standard deviation (SD) was 1.29 mg/cm^3^, and 95% limits of agreement ranged from −2.63 to 2.41. The vast majority of the difference in BMD values measured by normal-dose and low-dose iCare QCT fell within the limits of agreement, suggesting that the two scan modes are in good agreement. Table 3 presents correlation coefficients (r) between measured and true BMD values.

**Table 3:** Correlation coefficients (r) between measured and true BMD values.

Comparison	r	95% CI	*P*
iCare normal vs. true BMD	0.99	0.98–0.99	<0.001
iCare low vs. true BMD	0.99	0.98–0.99	<0.001
Normal vs. low-dose iCare (r)	0.99	0.98–0.99	<0.001
Difference between correlations	–	–	0.203

BMD, bone mineral density; CI, confidence interval.

### 4.5 Radiation dose from different scan modes

The CTDIvol of the normal-dose QCT scan was 12.69–13.29 mGy, with the mean value of (13.05 ± 0.20) mGy; the DLP was 223.50–234.45 mGy·cm, with the mean value of (226.34 ± 4.11) mGy·cm. The CTDIvol of the low-dose QCT scan was 1.91 mGy; the DLP was 32.68–32.69 mGy·cm, with the mean value of 32.68 mGy-cm. The results indicated that the CTDIvol and DLP of low-dose QCT were significantly lower than those of normal-dose QCT, with a reduction in radiation dose by about 85%.

### 4.6 Coefficient of variation (CV, %) for repeated BMD measurements

The CVs for repeated BMD measurements were calculated to evaluate the reproducibility of both QCT systems under different scan modes. The CVs of Mindways QCT for L1–L3 were 5.14%, 2.83%, and 0.46%, respectively at normal-dose, and 9.04%, 4.67%, and 0.48%, respectively at low-dose. The CVs of iCare QCT for L1–L3 were 1.10%, 0.80%, and 0.44%, respectively at normal-dose, and 2.32%, 0.84%, and 0.32%, respectively at low-dose. These results indicated that iCare QCT yielded lower CVs across all vertebrae and scan modes, indicating superior repeatability compared with Mindways QCT. Additionally, CV values decreased with increasing BMD in both systems, consistent with trends observed for RSD. Table 4 presents coefficient of variation (CV, %) for repeated BMD measurements.

**Table 4:** Coefficient of variation (CV, %) for repeated BMD measurements.

Vertebra	Mindways (normal)	Mindways (low)	iCare (normal)	iCare (low)
L1	5.14%	9.04%	1.10%	2.32%
L2	2.83%	4.67%	0.80%	0.84%
L3	0.46%	0.48%	0.44%	0.32%

BMD, bone mineral density.

## 5 Discussion

Accurate measurement of BMD is essential for evaluating bone quality and guiding treatment decisions. While clinical concerns, such as osteoporosis diagnosis and fracture risk are primary motivations for BMD assessment, phantom-based validation of imaging systems plays a crucial role in ensuring measurement fidelity and reproducibility (8, 9). This study aimed to evaluate the accuracy and precision of BMD measurements under normal-dose and low-dose QCT conditions using a standardized phantom.

Mindways QCT, a classic BMD assessment system, is widely used clinically for BMD measurement (12, 13, 15, 16) and is used as control to evaluate the accuracy of measured BMD values from different brands of QCT (5, 12). Previous studies (17–19) have shown that low-dose Mindways QCT is sensitive for screening of osteoporosis and osteopenia, but our previous studies have found that there were some errors in BMD measurements by either conventional normal-dose or low-dose Mindways QCT. The iCare QCT bone densitometry system is a newly developed system for measuring spine and hip BMD. Liu et al. (5) conducted a study in 131 patients who underwent QCT scanning of the hip and found that, by assessing bone mass in the hip, normal-dose iCare QCT had a higher detection rate of osteoporosis than the Mindways QCT. Our previous studies have also found that iCare QCT was superior to the Mindways QCT in measuring vertebral BMD, but whether there is a difference between vertebral BMD measurements by low-dose iCare QCT and Mindways QCT has not been reported (20, 21). The purpose of this study was to evaluate the accuracy and precision of BMD measurements by low-dose iCare QCT using the true ESP values and measured values by low-dose Mindways QCT for comparison.

Previous studies (22) indicated that BMD values in middle-aged and elderly people mostly fall into the category of osteoporosis and reduced bone mass. Therefore, during the evaluation of BMD measurement methods, special attention should be paid to their accuracy in populations with vertebral osteoporosis and/or osteopenia. In this study, we found that the RMEs of both normal-dose and low-dose iCare QCT measurements for L1 and L2 were less than 5%. The RMEs of both normal-dose and low-dose iCare QCT measurements for L3 were less than 9%. In addition, the BMD values of L1–L3 measured by normal-dose and low-dose iCare QCT were highly correlated with the true values and the correlation coefficients between them showed no statistically significant difference, which meets the clinical requirements for BMD measurements. The observed variability in BMD values may also reflect differences in the automatic ROI selection algorithms embedded within each QCT software system. Automatic ROIs, while time-efficient and reproducible, can be sensitive to segmentation parameters and edge-detection thresholds, especially when distinguishing cortical from trabecular bone in mid-density regions. Subtle shifts in ROI placement, particularly in phantoms with uniform inserts, might amplify impact on measurement values, especially at intermediate BMD levels, such as L2. This suggests the need for software optimization or manual override options in cases where measurement accuracy is critical. This study demonstrated significant differences (*P* < 0.050) between iCare QCT and Mindways QCT measurements at either normal-dose or low-dose. Compared with Mindways QCT, the BMD values of L1 and L2 measured by normal-dose and low-dose iCare QCT had smaller RMEs, suggesting better accuracy. Although iCare QCT did not show marked superiority in accuracy of L3 BMD measurement, its clinical application value was not impacted. Therefore, compared with Mindways QCT, normal-dose and low-dose iCare QCT is more favorable for the early diagnosis and treatment of patients with osteoporosis and osteopenia, and has more guiding significance and greater value for clinical application.

In terms of measurement precision, the relative standard deviation (RSD) of L1–L3 vertebral measurements obtained using both QCT software programs decreased as BMD increased. This trend indicates greater variability and precision error in the assessment of low bone density, aligning with findings reported in previous studies (5, 23). In addition, the RSDs of L1–L3 vertebrae measurements by normal-dose and low-dose iCare QCT were smaller than those of the Mindways QCT, indicating that iCare QCT had better precision and reproducibility than Mindways QCT in measuring vertebral BMD at either normal-dose or low-dose.

In this study, the radiation dose was reduced by decreasing the tube current, resulting in significantly lower CTDIvol and DLP values for the low-dose QCT compared to the normal-dose protocol. This reduction corresponded to an approximately 85% decrease in radiation exposure relative to the standard protocol, thereby markedly improving the clinical feasibility of routine QCT-based osteoporosis screening, particularly in populations requiring repeated imaging. There was no significant difference between normal-dose and low-dose Mindways QCT measurements (*P* > 0.050), which is consistent with the results of previous studies (17–19). There was no significant difference between normal-dose and low-dose iCare QCT measurements (*P* > 0.050), and there was a high level of correlation and agreement between the measured values, suggesting that low-dose iCare QCT significantly reduced the radiation dose without compromising the accuracy of the measurement. A possible explanation is that the tube current determines the number of x-ray photons but does not affect the x-ray penetration. A previous study (24) demonstrated that when the tube voltage is held constant, variations in tube current alone do not influence the x-ray attenuation coefficient of materials and thus do not alter the resulting CT values. Quantitative computed tomography (QCT) bone densitometry is a technique that utilizes standard CT scan data, whereby the CT values of the lumbar spine or hip are linearly regressed and converted into volumetric bone mineral density (BMD) using dedicated software and a calibration phantom with known density (25). Therefore, changing the tube current alone had no significant effect on vertebral BMD measurement by QCT.

Compared with DXA, QCT enables three-dimensional volumetric assessment of bone mineral density (BMD) and is not affected by degenerative changes, abdominal aortic calcifications, or variations in body size. In contrast, DXA measures areal BMD and may overestimate bone density in the presence of osteophytes or other degenerative alterations. Although DXA remains the primary tool in numerous clinical settings due to its lower cost and minimal radiation exposure, its limited specificity has led to growing interest in QCT, particularly for research purposes and precision monitoring. The phantom-based design of the present study allowed for direct comparison between QCT systems but did not include a DXA comparison. Incorporating such comparisons in future studies would provide additional insight into the relative strengths of each modality for diagnostic and longitudinal monitoring purposes.

In this study, the COV (expressed as RSD%) was also analyzed to assess the precision of repeated BMD measurements. The RSDs of Mindways QCT measurements ranged from 0.47% to 5.16% at normal-dose and from 0.49% to 9.08% at low-dose, while those of iCare QCT ranged from 0.45% to 1.11% at normal-dose and from 0.33% to 2.34% at low-dose. These findings indicate that iCare QCT demonstrated consistently lower RSDs across all lumbar vertebrae compared to Mindways QCT under both scan modes. A lower RSD indicates better repeatability, suggesting that iCare QCT provides more stable and reliable BMD measurements, even when operated in a low-dose mode. This high degree of precision further supports the suitability of iCare QCT for routine clinical application, particularly in settings where reducing radiation exposure is a priority. In this study, 10 repeated measurements were performed to assess precision. Although this number is considered adequate based on the known structural homogeneity of the phantom and consistent positioning, it may not capture long-term or inter-operator variability. Future studies involving physical repositioning between scans or using anthropomorphic phantoms may help to more comprehensively assess repeatability in clinically relevant scenarios. Additionally, small misalignments in phantom orientation or mechanical wear over time can subtly perturb measurements despite controlled scanning parameters, highlighting the importance of standardized scanning setups and phantom maintenance in longitudinal assessments.

With the improvement of public health awareness and the widespread availability of CT scanners across medical institutions in China, low-dose chest CT has been increasingly used for routine physical examinations and thoracic disease screening. Previous research (26) has demonstrated the feasibility of integrating low-dose chest CT with Mindways QCT for vertebral BMD assessment in a one-stop scanning program. In this study, the potential application of low-dose iCare QCT in a similar context was evaluated using a phantom model. Although the findings support the accuracy and precision of low-dose iCare QCT for BMD measurement at L1–L3, the study was limited to a phantom and did not include thoracic structures or clinical patients. Therefore, while the results suggest promise for combined thoracic screening and bone densitometry using low-dose iCare QCT, further clinical research is necessary to validate its utility in detecting thoracic diseases and to assess performance across a broader anatomical range.

Previous research has demonstrated that both iCare QCT and Mindways QCT effectively assess BMD in the lumbar spine. A study by Mont et al. (27) found no significant differences between the two systems in evaluating the ESP vertebral bodies (L1–L3), indicating comparable accuracy in BMD measurements. It is noteworthy that significant differences in spine BMD measurements have been observed between different CT scanner brands and models. A multicenter study (16) highlighted the necessity of cross-calibration in multi-center studies to ensure measurement consistency. Concerns regarding radiation exposure in QCT procedures have been addressed in prior studies. For instance, a study by Damilakis et al. (28) reported that optimized QCT protocols could achieve effective doses ranging from 0.06 to 0.3 mSv, which is significantly lower than doses associated with high-resolution CT imaging. Implementing low-dose QCT protocols has been shown to substantially reduce radiation exposure without compromising image quality. Museyko et al. (29) developed a low-radiation exposure protocol for 3D QCT of the spine, achieving dose reductions while maintaining diagnostic accuracy. Ensuring the reproducibility of BMD measurements over time is crucial for monitoring osteoporosis. A study by du Mont et al. (30) evaluated the long-term reproducibility of clinical QCT using different calibration methods and protocols, highlighting the importance of standardized procedures. Lin et al. (31) compared the detection rate of lumbar osteoporosis between QCT and DXA and found that QCT provided a more accurate evaluation of lumbar osteoporosis than DXA. Other scholars (32) compared DXA and QCT to determine their sensitivity and discriminatory power. They demonstrated that volumetric measurements by QCT in preselected subjects represented a more sensitive method for the diagnosis of osteoporosis and prediction of fractures compared with DXA. Wang et al. (33) assessed the parameter of dual-energy spectral CT (DesCT) consistency with BMD determination using QCT, and found that BMD values measured by DesCT were stable and repeatable under different radiation doses. DesCT and QCT measurements of human BMD were highly correlated (33).

In the present study, although iCare QCT generally exhibited lower RMEs compared with Mindways QCT across most vertebral levels, the RME for the L2 vertebra was slightly higher with iCare QCT under both scanning conditions. This deviation could be attributed to several factors. Firstly, L2 represented an intermediate bone density (osteopenia), which might inherently present more variability in detection thresholds for segmentation and calibration algorithms. Secondly, the software-specific ROI modeling and edge detection algorithms might respond differently to the moderate-density insert, especially in distinguishing trabecular from cortical boundaries. Thirdly, given the uniform structure of the phantom, slight variations in alignment or ROI positioning during automated processing, particularly in the mid-density insert, might have a more remarkable effect on measurement accuracy. Fourthly, this discrepancy did not appear to be due to scanning parameters, as all conditions were controlled and standardized across both QCT systems. Further investigation into the specific image processing approaches and calibration curve behaviors of each software package may clarify the origin of this localized error pattern.

## 6 Limitations

There are several limitations in this study. Firstly, this study was based on ESP, which did not include the effects of factors such as abdominal fat and ribs, and the study results need to be further verified clinically. Secondly, only GE revolution CT was used for BMD measurements and no multicenter comparisons were made between different brands and models of equipment. Thirdly, this study used 10 repeated scans per group, which is standard in phantom studies due to the high stability and lack of biological variability. Nonetheless, we acknowledge that a larger number of repetitions could improve the statistical confidence of RSD estimates. Future studies will include more repeated scans and confidence intervals for additional parameters to further enhance the robustness of the findings. Another key limitation of this study is that the data were obtained from ESP phantom measurements rather than clinical patients. While the phantom model ensures standardized conditions for evaluating measurement accuracy and precision, it cannot fully replicate the anatomical and physiological complexities present *in vivo*. Fifthly, the current evaluation was limited to the GE Revolution CT platform. Given that scanner-specific factors, such as beam hardening, reconstruction algorithms, and detector characteristics can influence quantitative measurements, further cross-platform validation studies are necessary to determine the reliability and transferability of iCare QCT performance on a broader range of CT systems. Finally, although the ESP provided a controlled and standardized environment for initial technical evaluation, its lack of anatomical complexity could limit the generalizability of results to clinical populations. Future studies will include real human subjects to further verify the accuracy and reliability of low-dose iCare QCT in clinical settings, as well as involving clinical patient data are necessary to validate the findings and assess the diagnostic performance of iCare QCT and Mindways QCT in real-world settings. Incorporating clinical BMD measurements across diverse populations will provide more comprehensive evidence of the applicability and reliability of low-dose QCT protocols in routine practice.

## 7 Conclusion

In summary, this phantom-based study demonstrated that iCare QCT provides higher precision in BMD measurements of the European Spine Phantom compared to Mindways QCT under both normal- and low-dose conditions. These findings support the broader application of low-dose QCT in clinical settings where radiation reduction and cost-effectiveness are priorities.

## Data Availability

The datasets presented in this study can be found in online repositories. The names of the repository/repositories and accession number(s) can be found in the article/Supplementary Material.
